# Supplementary material for: KIT ligand protects against both light-induced and genetic photoreceptor degeneration
Source: eLife. 2020 Apr 3;9:e51698. doi: 10.7554/eLife.51698 (PMC7170656; doi:10.7554/eLife.51698)
Supplement: Supplementary file 1. [file elife-51698-supp1.doc]

**Supplemental File 1**

| Key Resources Table | | | | |
| --- | --- | --- | --- | --- |
| Reagent type (species) or resource | Designation | Source or reference | Identifiers | Additional information |
| gene (*M. musculus*) | *Kitl* | NCBI database | NM_013598.3 |  |
| gene (*M. musculus*) | *Kit* | NCBI database | NM_001122733.1 |  |
| Strain (*M. musculus*) | C57BL/6J | Jackson Laboratory | Cat#: 000664  RRID: MGI:3028467 |  |
| Strain (*M. musculus*) | Balb/c | Charles river | Cat#: 211  RRID: MGI:2161072 | **Vital River Laboratory (Beijing, China)** |
| Strain (*M. musculus*) | *Kit^Wps/Wps^* | Gift from Drs. Xiang Gao, Wei Li | RRID: MGI:5523955 |  |
| Strain (*M. musculus*) | *rd1/rd1*  (FVB(ICR)-Pde6b^rd1^/Daiw) | Charles river | Cat#: 215  RRID: MGI:6353933 | **Vital River Laboratory (Beijing, China)** |
| Strain (*M. musculus*) | *rd10/rd10*  (B6.CXB1-Pde6b^rd10^/J) | Jackson Laboratory | Cat#: 004297  RRID: MGI:2173852 |  |
| Cell line (*M. musculus*) | 661W | Gift from Dr. Muayyad Al-Ubaidi, | RRID:CVCL_6240 | 661 W cell line tested negative for mycoplasma contamination by the MycoProbe Mycoplasma detection Kit and positive for photoreceptor cell marker Recoverin and Opsin by IF. |
| Biological sample (*M. musculus*) | Neural retina | Freshly isolated in lab |  | See Materials and Methods of this paper |
| Biological sample (*M. musculus*) | RPE | Freshly isolated in lab |  | See Materials and Methods of this paper |
| Biological sample (*M. musculus*) | Photoreceptor tissue | Freshly isolated in lab |  | See Materials and Methods of this paper |
| Peptide, recombinant protein | Recombinant Human KITL | R&D systems | Cat#: 255-SC |  |
| Peptide, recombinant protein | Q5 High-Fidelity DNA Polymerase | New England Biolabs | Cat#: M0493S |  |
| Antibody | Anti-GFAP (Rabbit polyclonal) | Abcam | Cat#: ab7260  RRID:AB_305808 | 1:500 (IF) |
| Antibody | Anti-KIT (Rabbit polyclonal) | Abcam | Cat#: ab5506  RRID:AB_91654 | 1:100 (IF)  1:800 (WB) |
| Antibody | Anti-NRF2 (Rabbit polyclonal) | Abcam | Cat#: AB137550  RRID:AB_2687540 | 1:200 (IF)  1:1000 (WB) |
| Antibody | Anti- Rhodopsin (Mouse monoclonal) | Millipore | Cat#: MABN15  RRID:AB_10807045 | 1:200 (IF) |
| Antibody | Anti-Opsin (Rabbit polyclonal) | Millipore | Cat#: AB5745  RRID:AB_240810 | 1:200 (IF) |
| Antibody | Anti-OTX2 (Rabbit polyclonal) | Millipore | Cat#: AB9566  RRID:AB_2157186 | 1:200 (IF) |
| Antibody | Anti-PKCα (Mouse monoclonal) | Santa Cruz Biotechnology | Cat#: sc-8393  RRID:AB_628142 | 1:50 (IF) |
| Antibody | Anti-HMOX1 (Rabbit polyclonal) | Santa Cruz Biotechnology | Cat#: sc-10789  RRID:AB_648281 | 1:50 (IF)  1:200 (WB) |
| Antibody | Anti-p-KIT (Tyr568/570) (Rabbit polyclonal) | Santa Cruz Biotechnology | Cat#: sc-18076-R  RRID:AB_653087 | 1:1000 (WB) |
| Antibody | Anti-α-Tubulin (Mouse monoclonal) | Santa Cruz Biotechnology | Cat#: sc-53646  RRID:AB_630403 | 1:1000 (WB) |
| Antibody | Anti-β-Actin (Mouse monoclonal) | Santa Cruz Biotechnology | Cat#: sc-130300  RRID:AB_2223499 | 1:1000 (WB) |
| Antibody | IRDye® 800CW Donkey anti-mouse lgG(H+L) | LI-COR | C70502-02 | 1:5000 (WB) |
| Antibody | IRDye® 680RD Donkey anti-rabbit lgG(H+L) | LI-COR | C30420-01 | 1:5000 (WB) |
| Antibody | IRDye® 800CW Donkey anti-goat lgG(H+L) | LI-COR | C11117-05 | 1:5000 (WB) |
| Antibody | IRDye® 800CW Goat anti-rat lgG(H+L) | LI-COR | C11024-01 | 1:5000 (WB) |
| Antibody | IRDye® 680RD Goat anti-mouse lgG(H+L) | LI-COR | C30502-01 | 1:5000 (WB) |
| Antibody | Anti-KIT (ACK45) (Rat monoclonal) | BD Pharmingen | Cat#: 553868  RRID:AB_395102 | 1:50 (IF)  1:200 (WB) |
| Antibody | Anti-KIT (ACK2 / CD117) (Rat monoclonal) | Invitrogen | Cat#: 14-1172-82  RRID:AB_467436 | 1:50 (IF) |
| Antibody | Alexa 594-conjugated donkey anti-rabbit lgG(H+L) | Invitrogen | Cat#: 1827674 | 1:500 (IF) |
| Antibody | Alexa 488-conjugated donkey anti-mouse lgG(H+L) | Invitrogen | Cat#: 1796361 | 1:200 (IF) |
| Antibody | Alexa 594-conjugated donkey anti-mouse lgG(H+L) | Invitrogen | Cat#: 1820027 | 1:500 (IF) |
| Antibody | Alexa 488-conjugated donkey anti-rabbit lgG(H+L) | Invitrogen | Cat#: 1834802 | 1:200 (IF) |
| Antibody | Alexa 488-conjugated donkey anti-goat lgG(H+L) | Invitrogen | Cat#: 1605893 | 1:200 (IF) |
| Antibody | Anti-KITL/SCF (Goat polyclonal) | R&D systems | Cat#: AF-455-NA  RRID:AB_2265325 | 1:50 (IF)  1:800 (WB) |
| Antibody | Anti-p44/42 MAPK (Erk1/2) (Rabbit polyclonal) | Cell signaling technology | Cat#: 4695S  RRID:AB_390779 | 1:2000 (WB) |
| Antibody | Anti-p-p44/42 MAPK (Erk1/2) (Thr202/Tyr204) (Rabbit polyclonal) | Cell signaling technology | Cat#: 4370S  RRID:AB_2315112 | 1:2000 (WB) |
| Antibody | Anti- AKT (Rabbit polyclonal) | Cell signaling technology | Cat#: 9272S  RRID:AB_329827 | 1:2000 (WB) |
| Antibody | Anti-p-AKT  (Rabbit polyclonal) | Cell signaling technology | Cat#: 4060S  RRID:AB_2315049 | 1:2000 (WB) |
| Antibody | Anti-Flag (Rabbit polyclonal) | Cell signaling technology | Cat#: 14793S  RRID:AB_2572291 | 1: 200 (IF)  1:2000 (WB) |
| Antibody | Anti-EAAT1 (Goat polyclonal) | Santa Cruz Biotechnology | Cat#: sc-7757  RRID:AB_2190593 | 1: 200 (IF) |
| Recombinant DNA reagent | p-rAAV2-RHO-KITL | Made in lab |  | See Materials and Methods of this paper |
| Recombinant DNA reagent | p-rAAV2-RPE65-KITL | Made in lab |  | See Materials and Methods of this paper |
| Recombinant DNA reagent | p-rAAV2-CMV empty vector | Genechem, China |  |  |
| Recombinant DNA reagent | p-rAAV2-RPE65 empty vector | Genechem, China |  |  |
| Recombinant DNA reagent | p-rAAV2-CMV-KITL | Made in lab |  | See Materials and Methods of this paper |
| Recombinant DNA reagent | p-rAAV2-CMV-HMOX1 | Made in lab |  | See Materials and Methods of this paper |
| Sequence-based reagent | Genotyping  Murine Kit^Wps^  Forward | Tsingke  China |  | 5’-TTGACCCAGA  GAAAGCTGTG-3’ |
| Sequence-based reagent | Genotyping  Murine Kit^Wps^  Reverse | Tsingke  China |  | 5’-GAGAACAGGG  AAGCAACTACC-3’ |
| Sequence-based reagent | Genotyping  Murine Rpe65  Forward | Tsingke  China |  | 5’-CTGACAAG  CTCTGTAAG-3’ |
| Sequence-based reagent | Genotyping  Murine Rpe65  Reverse | Tsingke  China |  | 5’-CATTACCATC  ATCTTCTTCCA-3’ |
| Sequence-based reagent | Subcloning  Murine *Hmox1*  Forward | Tsingke  China |  | 5’-GGCATGCCTTC  CGCATACAACCAGTG-3’ |
| Sequence-based reagent | Subcloning  Murine *Hmox1*  Reverse | Tsingke  China |  | 5’-AGGCGCGCCT  TCAGGTATC  TCCCTCCATT-3’ |
| transfected construct (*M. musculus*) | siRNA to Nrf2 line 1 | GenePharma  China |  | 5’-GCAGGACAUGGAUUUGAUUTT-3’ / 5’-AAUCAAAUCCAUGUCCUGCTT-3’ |
| transfected construct (*M. musculus*) | siRNA to Nrf2 line 2 | GenePharma  China |  | 5’-CCGAAUUACAGUGUCUUAATT-3’ / 5’-UUAAGACACUGUAAUUCGGTT-3’ |
| transfected construct (*M. musculus*) | siRNA to Kitl line 1 | GenePharma  China |  | 5’-GCUCCUAUUUAAUCCUCUUTT-3’ / 5’-AAGAGGAUUAAAUAGGAGCTT-3’ |
| transfected construct (*M. musculus*) | siRNA to Kitl line 2 | GenePharma  China |  | 5’-GCUACGAGAUAUGGUAAUATT-3’ / 5’-UAUUACCAUAUCUCGUAGCTT-3’ |
| transfected construct (*M. musculus*) | siRNA to Hmox1 line 1 | GenePharma  China |  | 5’-CCACCAAGGAGGUACACAUT-3’ / 5’-AUGUGUACCUCCUUGGUGGTT-3’ |
| transfected construct (*M. musculus*) | siRNA to Hmox1 line 2 | GenePharma  China |  | 5’-GCUGACAGAGGAACACAAATT-3’ / 5’-UUUGUGUUCCUCUGUCAGCTT-3’ |
| transfected construct (*M. musculus*) | siRNA NC | GenePharma  China |  | 5’-UUCUCCGAACGUGUCACGUTT-3’ / 5’-ACGUGACACGUUCGGAGAATT-3’ |
| Sequence-based reagent | Amplifying Murine Gapdh  Forward | TsingKe  China | Gene ID: 14433 | 5’-ACCACAGTCCATGCCATCAC-3’ |
| Sequence-based reagent | Amplifying Murine Gapdh  Reverse | TsingKe  China | Gene ID: 14433 | 5’-TCCACCACCCTGTTGCTGTA-3’ |
| Sequence-based reagent | Amplifying Murine Lif  Forward | TsingKe  China | Gene ID: 16878 | 5’-AACGATGGTGTCACCCTGAAG-3’ |
| Sequence-based reagent | Amplifying Murine Lif  Reverse | TsingKe  China | Gene ID: 16878 | 5’-TATATACTGGAGCCGTGGTCT-3’ |
| Sequence-based reagent | Amplifying Murine Relb  Forward | TsingKe  China | Gene ID: 19698 | 5’-TAGCCTTGCACCGCTTGC-3’ |
| Sequence-based reagent | Amplifying Murine Relb  Reverse | TsingKe  China | Gene ID: 19698 | 5’-CAATTCATCTGTGGTCCTGGAGAC-3’ |
| Sequence-based reagent | Amplifying Murine Epha2  Forward | TsingKe  China | Gene ID: 13836 | 5’-GGTTCTCACCCAACTTCCAT-3’ |
| Sequence-based reagent | Amplifying Murine Epha2  Reverse | TsingKe  China | Gene ID: 13836 | 5’-CGAAGTCCAACAAAACAACTTCCT-3’ |
| Sequence-based reagent | Amplifying Murine c-fos  Forward | TsingKe  China | Gene ID: 14281 | 5’-CAGAGCGGGAATGGTGAAGA-3’ |
| Sequence-based reagent | Amplifying Murine c-fos  Reverse | TsingKe  China | Gene ID: 14281 | 5’-CTGTCTCCGCTTGGAGTGTA-3’ |
| Sequence-based reagent | Amplifying Murine Nfkb1  Forward | TsingKe  China | Gene ID: 18033 | 5’-GGTCACCCATGGCACCATAA-3’ |
| Sequence-based reagent | Amplifying Murine Nfkb1  Reverse | TsingKe  China | Gene ID: 18033 | 5’-AGCTGCAGAGCCTTCTCAAG-3’ |
| Sequence-based reagent | Amplifying Murine Ngf  Forward | TsingKe  China | Gene ID: 18049 | 5’-GGAGCGCATCGAGTGACTT-3’ |
| Sequence-based reagent | Amplifying Murine Ngf Reverse | TsingKe  China | Gene ID: 18049 | 5’-CCTCACTGCGGCCAGTATAG-3’ |
| Sequence-based reagent | Amplifying Murine Myd88  Forward | TsingKe  China | Gene ID: 17874 | 5’-TGTTCTTGAACCCTCGGACG-3’ |
| Sequence-based reagent | Amplifying Murine Myd88  Reverse | TsingKe  China | Gene ID: 17874 | 5’-TTCTGGCAGTCCTCCTCGAT-3’ |
| Sequence-based reagent | Amplifying Murine Hmox1  Reverse | TsingKe  China | Gene ID: 15368 | 5’-CAGAAGAGGCTAAGACCGCC-3’ |
| Sequence-based reagent | Amplifying Murine Hmox1  Forward | TsingKe  China | Gene ID: 15368 | 5’-GCAGTATCTTGCACCAGGCTA-3’ |
| Sequence-based reagent | Amplifying Murine Nfe2l2  Reverse | TsingKe  China | Gene ID: 18024 | 5’-CATGAGACATCTGCTCGCCT-3’ |
| Sequence-based reagent | Amplifying Murine Nfe2l2  Forward | TsingKe  China | Gene ID: 18024 | 5’-ACTGCCCAAGCAGAGTTAGG-3’ |
| Sequence-based reagent | Amplifying Murine Kit  Reverse | TsingKe  China | Gene ID: 16590 | 5’-AAGCGTCTCCACCATTCA-3’ |
| Sequence-based reagent | Amplifying Murine Kit  Forward | TsingKe  China | Gene ID: 16590 | 5’-GGAGCGGTCAACAAGGAA-3’ |
| Sequence-based reagent | Amplifying Murine Kitl V1  Reverse | TsingKe  China | Gene ID: 17311 | 5’-TTATGTTACCCCCTGTTGCAG-3’ |
| Sequence-based reagent | Amplifying Murine Kitl V1  Reverse | TsingKe  China | Gene ID: 17311 | 5’-CTGCCCTTGTAAGACTTGACTG-3’ |
| Sequence-based reagent | Amplifying Murine Kitl V2  Forward | TsingKe  China | Gene ID: 17311 | 5’-TCCCGAGAAAGGGAAAGC-3’ |
| Sequence-based reagent | Amplifying Murine Kitl V2  Reverse | TsingKe  China | Gene ID: 17311 | 5’-CTGCCCTTGTAAGACTTGACTG-3’ |
| Sequence-based reagent | Amplifying Murine Rpe65  Forward | TsingKe  China | Gene ID: 17342 | 5’-CACTCTGCGCAGTGATGAGA-3’ |
| Sequence-based reagent | Amplifying Murine Rpe65  Reverse | TsingKe  China | Gene ID: 17342 | 5’-CTTACAGAGCTTGTCAGGAACA-3’ |
| Sequence-based reagent | Amplifying Murine Mitf  Reverse | TsingKe  China | Gene ID: 19892 | 5’-GCAAGAGGGAGTCATGCAGT-3’ |
| Sequence-based reagent | Amplifying Murine Mitf  Reverse | TsingKe  China | Gene ID: 19892 | 5’-AGAACTGCTGCTCTTCAGAGGT-3’ |
| Commercial assay, kit | In Situ Cell Death Detection Kit | Roche | Cat#: 11684795910 |  |
| Commercial assay, kit | cell fractionation kit-standard | Abcam | Cat#: ab109719 |  |
| Software | Image J | NIH | SCR:003070 |  |
| Software | Photoshop | Adobe | SCR:014199 |  |
| Software | SPSS | SPSS | SCR:002865 |  |
